# Methyltransferase DNMT3B promotes colorectal cancer cell proliferation by inhibiting PLCG2: DNMT3B promotes CRC cell proliferation by inhibiting PLCG2

**DOI:** 10.3724/abbs.2024117

**Published:** 2024-08-07

**Authors:** Yong Ji, Yang Wang, Jiacheng Zou, Guanghao Liu, Mingyu Xia, Jun Ren, Daorong Wang

**Affiliations:** 1 The Yangzhou School of Clinical Medicine of Dalian Medical University Yangzhou 225001 China; 2 Clinical Medical College Yangzhou University Yangzhou 225001 China; 3 Jingjiang People’s Hospital Jingjiang 214500 China; 4 The Yangzhou Clinical Medical College of Xuzhou Medical University Yangzhou 225001 China; 5 General Surgery Institute of Yangzhou Yangzhou University Yangzhou 225001 China; 6 Yangzhou Key Laboratory of Basic and Clinical Transformation of Digestive and Metabolic Diseases Yangzhou 225001 China

**Keywords:** epigenetics, DNA methylation, DNMT3B, PLCG2, colorectal cancer

## Abstract

Aberrant DNA methylation patterns in the promoter region of *PLCG2* are associated with dysregulated signaling pathways and cellular functions. Its role in colorectal cancer cells is still unknown. In this study, qRT-PCR is used to measure *DNMT3B* expression in colorectal cancer. Western blot analysis and immunohistochemistry are used to analyze DNMT3B and PLCG2 protein levels in colorectal tissues and cell lines. Cell Counting Kit-8 (CCK-8) and colony formation assays are used to assess the proliferation of colorectal cancer cells. Methylation-specific PCR (MSP) and bisulfite-sequencing PCR (BSP) are used to measure DNA methylation level. Our results show that DNMT3B is overexpressed in colorectal cells in the TCGA datasets according to Kaplan-Meier plots. DNMT3B is significantly overexpressed in tumor tissues compared to that in adjacent nontumor tissues. Western blot analysis results demonstrate high expression of DNMT3B in tumor tissues. Compared to normal colonic epithelial cells, colorectal cancer cell lines exhibit elevated level of *PLCG2* methylation. Overexpression of PLCG2 effectively prevents the growth of colorectal cancer xenograft tumors *in vivo*. PLCG2 is identified as a key downstream regulatory protein of DNMT3B in colorectal cancer. DNMT3B inhibits *PLCG2* transcription through methylation of the *PLCG2* promoter region. DNMT3B controls colorectal cancer cell proliferation through PLCG2, which is useful for developing therapeutic approaches that target PLCG2 expression for the treatment of colorectal cancer.

## Introduction

Colorectal cancer (CRC) is the third most common and deadly tumor type worldwide, although great progress has been made in CRC treatment over the past few years [1]. Although there have been some advancements in early diagnosis, the current surgical and chemoradiotherapy treatments are not optimal [2]. This is attributed to the pathological stage, invasion, and metastasis of CRC cells, which compromise the effectiveness of the treatment [3].

DNA methylation plays a crucial role in the initiation and progression of CRC [ 4‒ 6]. It is an epigenetic modification involving the addition of methyl groups to DNA molecules. In CRC, DNA methylation typically involves the increased or decreased methylation of certain genes, potentially leading to changes in the expression levels of these genes.

Generally, in CRC, tumor suppressor genes often undergo increased methylation, suppressing their function and diminishing their ability to resist cancer [7]. Conversely, some oncogenes may undergo decreased methylation, resulting in their overactivation and promoting the growth and spread of cancer cells [8]. Furthermore, the patterns and extent of DNA methylation can serve as markers for the diagnosis and prognosis of CRC [ 9‒ 11]. Therefore, gaining a deeper understanding of the role of DNA methylation in CRC not only helps unravel the molecular mechanisms of the disease but also provides crucial clues for the development of more effective treatment and diagnostic approaches.

DNA methyltransferase 3B (DNMT3B) is a DNA methyltransferase belonging to the DNA methyltransferase family. This enzyme is responsible for the addition of methyl groups to DNA molecules, thereby influencing gene expression and cellular genomic stability. Abnormal DNMT3B activity has been implicated in the occurrence and development of diverse cancers, specifically cervical cancer [12], breast cancer [13], endometrial cancer [14], and gastric cancer [15]. It induces abnormal DNA methylation, affecting the expression of tumor suppressor genes and oncogenes and consequently propelling tumor growth and advancement.

PLCG2, or phospholipase C-γ2, is a protein belonging to the phospholipase C enzyme family. It serves as a substrate for BTK (Bruton’s tyrosine kinase), and inactivation of BTK leads to the phosphorylation of AKT [16]. The interplay between PLCG2 and AKT phosphorylation underscores the complexity of cellular signaling networks and provides insights into potential mechanisms of negative feedback regulation in cellular processes. Phospholipase C-γ2 (PLCG2), recognized as a pivotal signaling protein, plays a vital role in maintaining normal cellular function and immune responses [17]. In-depth investigation into PLCG2 is instrumental in enhancing our understanding of its cellular biological functions and the underlying mechanisms of disease pathogenesis. This study not only contributes to unravelling the molecular intricacies of PLCG2 but also sheds light on its implications for cellular processes and disease mechanisms, providing valuable insights for potential therapeutics.

Understanding the molecular origins of CRC is essential for enhancing the survival rates of CRC patients. Consequently, a more comprehensive understanding of the molecular irregularities linked to CRC pathogenesis has the potential to enhance treatment modalities and improve the overall survival of individuals affected by this condition.

## Materials and Methods

### Cell culture

Human CRC cell lines (HCT116, SW620, COLO205 and RKO) and the epithelial cell line NCM460 were obtained from the Cell Bank of the Chinese Academy of Sciences (Shanghai, China). The RPMI-1640 medium (HyClone, Logan, USA) used for cell culture was supplemented with 1% penicillin/streptomycin and 10% fetal bovine serum (FBS; Life Technologies, Waltham, USA). The cells were cultivated at 37°C in a humidified incubator with 5% CO _2_.

### Patient samples

From March 2021 to July 2022, eighty patients with each set of (cancer and normal) frozen CRC tissues were obtained from CRC excision at the Northern Jiangsu People’s Hospital. CRC was confirmed at the time of initial diagnosis, and neither patient had previously undergone radiotherapy or chemotherapy. The samples taken from the patients were immediately placed in liquid nitrogen. This study was carried out according to the ethical approval obtained from the Medical Ethics Committee of Northern Jiangsu People’s Hospital (2020KY-137).

### Immunohistochemistry (IHC)

A tissue microarray (TMA) that included samples from 80 patients with histologically confirmed CRC and 80 controls was generated according to a previously described method [18]. The sections were deparaffinized in xylene, rehydrated in a graded alcohol series and citrate buffer, and then blocked with 3% hydrogen peroxide. Subsequently, the sections were incubated with a primary antibody directed against DNMT3B (57868, 1:100; Cell Signaling Technology, Beverly, USA) or PLCG2 (5690, 1:150; Cell Signaling Technology) and then with a biotin-conjugated secondary antibody (SA1050; Boster, Wuhan, China), followed by incubation with streptavidin-peroxidase complex (Boster). After color development, five high-power fields (400× magnification) were selected randomly and photographed for each slide. The protein expression score was evaluated by taking both the proportion of positive cells [0 (<5%), 1 (5%–25%), 2 (26%–50%), 3 (51%–75%), and 4 (>75%)] and the intensity of cell staining [0 (negative), 1 (weak), 2 (moderate), and 3 (strong)] into account. The final staining scores were calculated by multiplying the staining intensity by the degree of staining. DNMT3B staining was considered low or high using a cut-off value of 5 based on the analysis of the receiver operating characteristic (ROC) curve. A final score greater than 5 was defined as high expression of PLCG2.

### RNA-seq analysis

HCT1116 colon cancer cells were treated with 10 μM of SGI-1027 (a DNMT3B inhibitor sourced from MedChemExpres (Monmouth Junction, USA) for 24 h, while the control group was treated with an equivalent concentration of DMSO. Subsequently, RNA-seq analysis was performed on both groups by the BGI-Huada Genomics Institute (Wuhan, China). Clean reads were aligned to the reference gene sequences using Bowtie2, and then gene expression levels for each sample were calculated using RSEM. Differential expression analysis (DEA) of genes was conducted using the DESeq2 method, which is based on the negative binomial distribution principle. In this project, DEG (differentially expressed genes) identification was performed according to the methodology described in Michael *et al* [19]. The top 20 differentially expressed genes (DEGs) with fold change ≥2 or ≤–2 and a *P*-value ≤0.001 were selected for further analysis. Hierarchical clustering analysis of these selected DEGs was conducted using the R package pheatmap. The base function ‘phyper’ in R was utilized to calculate the *P*-value. Subsequently, we applied multiple testing correction to the *P*-values using the ‘qvalue’ package. Finally, a threshold of Qvalue (corrected *P*-value) was set at ‒0.05, and GO terms that met this criterion were defined as significantly enriched GO terms among the candidate genes.

### Quantitative real-time PCR (qRT-PCR):

Total RNA was extracted by utilizing Trizol (Invitrogen, Carlsbad, USA), and a cDNA synthesis kit (K1622; Thermo Fisher Scientific, Waltham, USA) was used to reverse transcribe 1 μg of the extracted RNA. Each RT-PCR procedure required 200 ng of cDNA in total. Quantitative real-time PCR (qRT-PCR) was conducted using 2× Universal SYBR Green Fast qPCR Mix (ABclonal, Wuhan, China). The following amplification procedure was used: 95°C for 3 min, followed by 40 cycles of 95°C for 5 s and 60°C for 30 s. The primer pair sequences used were as follows: *PLCG2* forward primer 5′-TCCACCACGGTCAATGTAGAT-3′, reverse primer 5′-CCCTGGGCGGATTTCTTTTAT-3′; *DNMT3B* forward primer 5′-AGGGAAGACTCGATCCTCGTC-3′, reverse primer 5′-GTGTGTAGCTTAGCAGACTGG-3′; and *GAPDH* forward primer 5′-ACGGATTTGGTCGTATTGGGCG-3′, reverse primer 5′-GCTCCTGGAAGATGGTGATGGG-3′. The 2 ^–ΔΔCt^ method was used to determine target gene expression level.

### Western blot analysis

Cells were lysed with RIPA buffer (Beyotime, Shanghai, China). Each specimen (30 mg of protein) was separated by SDS-PAGE immediately after the protein concentration was determined by BCA assay. The separated proteins were then electrotransferred onto 0.45-μm polyvinylidene difluoride membranes (Millipore, Billerica, USA). The membranes were subsequently blocked with 5% skim milk for 2 h at room temperature after being washed three times with TBST. The membranes were then incubated with the primary antibody at 4°C overnight. The membranes were then incubated with a horseradish peroxidase-conjugated secondary antibody (1:5000; ABclonal) for 2 h at room temperature after being washed three times with TBST. After being treated with a secondary antibody coupled to peroxidase, signals were detected utilizing an enhanced chemiluminescence substrate (Millipore). The intensities of the western blots were measured using Image J (NIH, Bethesda, USA), and the bands were analyzed using GraphPad Prism 8 (GraphPad Software, La Jolla, USA). The primary antibodies used in this study were as follows: rabbit anti-DNMT3B (1:200, 57868; Cell Signaling Technology), rabbit anti-PLCG2 (1:2000, 5690; Cell Signaling Technology), rabbit anti-p-AKT (1:2000, 4060; Cell Signaling Technology), rabbit anti-CyclinD1 (1:2000, 2922; Cell Signaling Technology), and mouse anti-GAPDH (1:5000; ABclonal).

### Cell transfection

We utilized lentivirus to induce the overexpression of PLCG2 in the HCT116 cell line and established stable CRC cell lines with elevated PLCG2 levels. Two modified lentiviral vectors, namely, a vector (lentivirus-EGFP-Puro) and PLCG2 (lentivirus-PLCG2-EGFP-Puro), were generated by GeneChem (Shanghai, China). Subsequently, 1×10 ^4^ cells were seeded into each well of a 24-well plate 12 h before viral infection. The lentivirus was introduced into each well for a 72-h period, followed by puromycin screening to confirm the establishment of stable cell lines. Fluorescence microscopy (Olympus, Tokyo, Japan) was used to detect the fluorescence signal in the cells. The process was performed in accordance with the outlined methodology.

### Cell Counting Kit-8 (CCK-8) assay

A single-cell suspension was produced by digesting the cells with 0.25% trypsin after two rounds of washing with PBS when they had reached 80% confluency. SW620 colon cancer cells were treated with either 10 μM SGI-1027 or 30 μM 3-NC (a PLCG2 inhibitor from MedChemExpres) for 24 h. Six parallel wells were set for the experiment, and the cells were seeded into a 96-well plate at a density of 1500 cells per well in 100 μL of medium. Ten microliters of CCK-8 solution (CK13; Dojindo, Shanghai, China) was added to each well after 24, 48, and 72 h of incubation. The optical density (OD) of each well was then determined using a microplate reader (Tecan, Shanghai, China) at a wavelength of 450 nm. Each experiment was conducted three times.

### Colony formation assay

To evaluate the ability of single cells to form colonies, 500 cells/well were added to 24-well plates in RPMI 1640 basic medium supplemented with 10% FBS. The cells were incubated for 14 days at 37°C. Afterward, the cells were washed, fixed with 4% paraformaldehyde, and stained with 0.5% crystal violet in 3% acetic acid (v/v) for 20 min at room temperature. Colonies were photographed and quantified under a light microscope (Olympus).

### Cell cycle assay

To analyze the impact of PLCG2 overexpression on the cell cycle of intestinal cancer cells, cells were seeded in 6-well plates at 2×10 ^5^ cells per well. Following a 48-h incubation, the cells underwent harvesting and were rinsed with chilled PBS. Subsequently, these cells were fixed via the addition of 1 mL of 75% ethanol and stored overnight in a ‒20°C freezer. In the subsequent step, the cells were washed and resuspended in PBS, and then treated with 10 μL of RNase A (10 mg/mL; ACROS Organics, Waltham, USA) for 30 min at 37°C. This was followed by a mixing process and resuspension in 500 μL PBS containing 5 μL each of PI (Beyotime, Shanghai, China) and Triton X-100 (ACROS Organics), with a further 30-min incubation in the dark. To assess the cell cycle phases across all treatment groups, a Flow Cytometer (MACSQuant Analyzer 10, Miltenyi Biotech, Germany) was employed, and data analysis was conducted using FlowJo software (FlowJo LLC; TreeStar, Ashland, USA).

### Methylation-specific PCR (MSP) and bisulfite sequencing PCR (BSP)

Online MetPrimer software ( http://www.urogene.org/methprimer) was used to analyze CpG islands and design primers for human *PLCG2* promoters for methylation-specific PCR (MSP) and bisulfite sequencing PCR (BSP). For MSP analysis of the human *PLCG2* primer, we used the methylated forward primer 5′-ATTTTCGAGTTTAGACGATTTTTTC-3′, the reverse primer 5′-CGCAATCCAAATATTTACCGTA-3′, the unmethylated forward primer 5′-TTTTGAGTTTAGATGATTTTTGT-3′ and the reverse primer 5′-ACCACAATCCAAATATTTACCATA-3′. The PCR control for input DNA (Input) was performed with the forward primer 5′-CCTGGCGGGTAATTGTGAAGA-3′ and the reverse primer 5′-CTGCGTGCCAAAGAAGAAACT-3′. After PCR amplification, the products were analyzed on a 2% agarose gel and observed under ultraviolet light, and densitometric analysis was performed using ImageJ software.

Bisulfite sequencing of the human *PLCG2* promoter was performed with an EpiArt DNA Methylation Bisulfite kit (Vazyme Biotech, Nanjing, China). The genomic DNA was amplified by PCR with the BSP forward primer 5′-TTAGGTTTTTTAAAGAGTTGGGATG-3′ and the reverse primer 5′-ACAAAAAAAATTCCCCAATATCATA-3′. The BSP amplification product was purified with an amplification product purification kit (Vazyme Biotech) and cloned and inserted into the pGEM-Tasy vector system (Promega, Madison, USA). The percentages of methylated cytosines relative to total cytosines were calculated.

### Xenotransplantation experiment

A total of 10 4-week-old BALB/c male nude mice (weight, 18–22 g) were purchased from GemPharmatech Co., Ltd. (Nanjing, China) and raised in a pathogen-free laminar flow cabinet throughout the experiments under the following conditions: controlled humidity (30%–40%), a constant temperature of 25°C, a 12/12-h light/dark cycle and free access to food and water. Ethical approval (approval No. 202111022) to perform the animal experiments was obtained from the Ethics Committee for Animal Experiments of Yangzhou University (Yangzhou, China). The experimental protocol was performed in accordance with the Laboratory Animal Guidelines for Ethical Review of Animal Welfare. Four-week-old male BALB/c nude mice were randomly divided into vector and oePLCG2 groups ( *n*=5 in each group). Under isoflurane inhalation anesthesia (1%–2%), ~1×10 ^6^ HCT116 cells of the stably transfected Vector/oePLCG2 strains resuspended in 100 μL of PBS were subcutaneously injected into the left armpits of the mice. The health and behavior of the mice were monitored every 2 days to determine whether they had difficulties in eating or drinking, unrelieved pain or distress without recovery. If the tumor reached 2000 mm ^3^, the animal was euthanized as a humane endpoint. The following formula was used to calculate the tumor volume (V) every week: V=(width ^2^×length)/2. Four weeks postinoculation, all the mice were sacrificed by cervical dislocation under anesthesia. The method of anesthesia used for the mice was CO _2_ asphyxiation (CO _2_ was introduced into the chamber at a rate of 40%–70% of the chamber volume per min to minimize distress). Dilated pupils were then used to verify death. Then, the tumors were removed and weighed.

### Statistical analysis

Statistical analysis was performed using GraphPad Prism (version 8.0). Quantitative data are presented as the mean±SD. Student’s *t* test was used to analyze differences in the mean of two samples. The associations between DNMT3B expression and clinicopathological characteristics were analyzed by the chi-square test (χ2). Patient survival was evaluated utilizing the Kaplan-Meier method. Counting and calculation of the area were performed with ImageJ (1.46r). All statistical tests were considered significant when *P* <0.05.

## Results

### DNMT3B is overexpressed in colorectal cells in TCGA datasets

**Figure 1:**
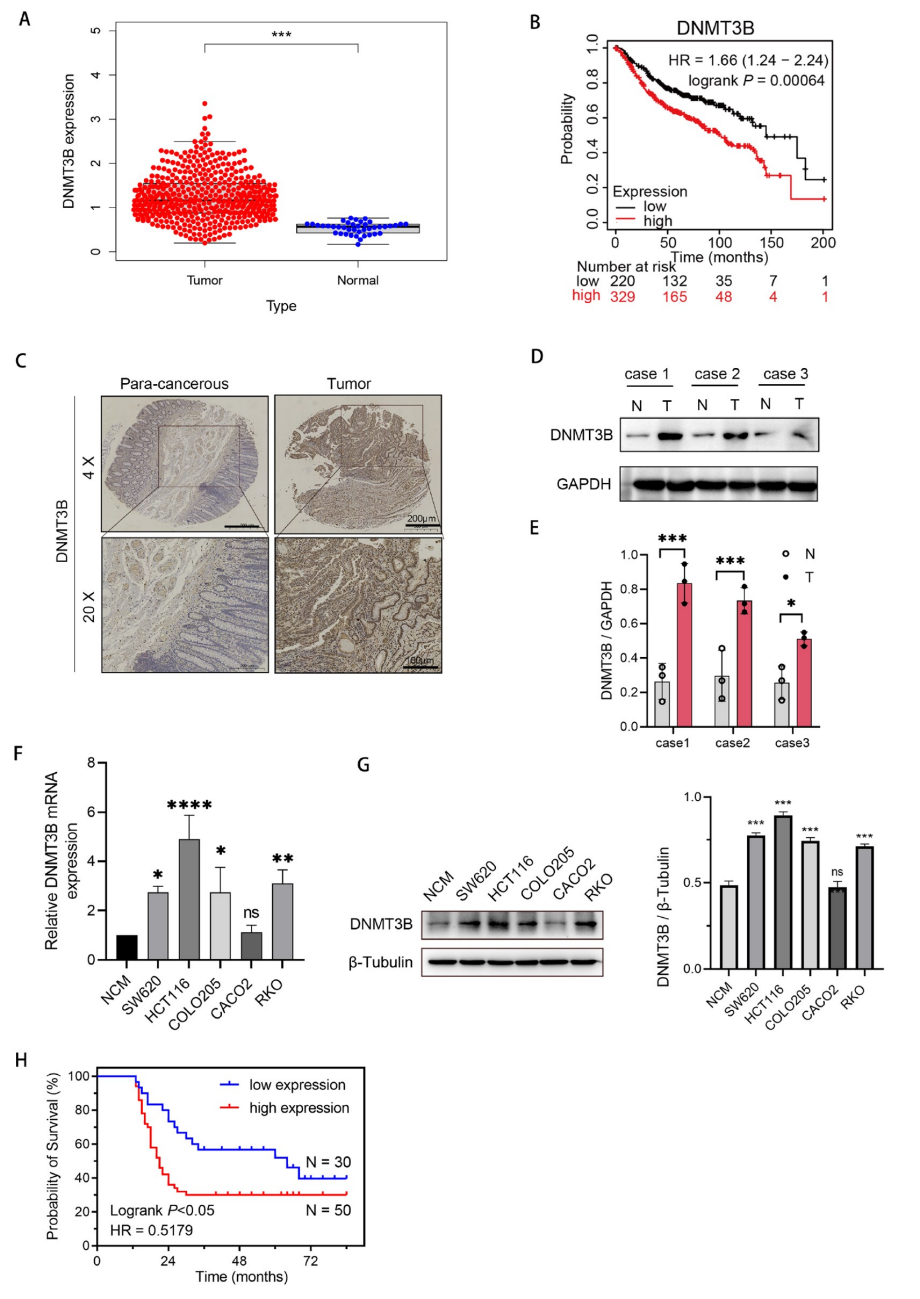
Figure 1 DNMT3B is overexpressed in colorectal cells in TCGA datasets (A) DNMT3B expression in colorectal cancer was analyzed via the TCGA database. (B) Kaplan-Meier plotter database was used to analyze the effect of DNMT3B on prognosis in colorectal cancer patients. (C) Immunohistochemical analysis of DNMT3B in paired noncancerous tissues and CRC tissues from patients. (D) The protein expression level of DNMT3B in paired noncancerous tissues and CRC tissues of patients. (E) Quantitative analysis of the western blots. (F) The mRNA expression level of DNMT3B in CRC cells and the NCM460 epithelial cell line measured by RT-qPCR. (G) Western blot analysis of the protein expression level of DNMT3B in CRC cells and the NCM460 epithelial cell line. (H) Kaplan-Meier survival analysis showed that DNMT3B overexpression predicted poorer prognosis in patients with CRC. NCM, NCM460. *P<0.05, **P<0.01, ***P<0.001, ****P<0.0001.

We found that DNMT3B is significantly overexpressed in tumor tissues in the TCGA database ( https://www.cancer.gov/ccg/research/genome-sequencing/tcga/using-tcga-data/citing) ( Figure 1A). Subsequently, we analyzed the effect of DNMT3B on survival using the Kaplan-Meier plotter database (kmplot.com). The results showed that patients with high DNMT3B expression exhibited poorer prognoses ( Figure 1B). To validate the expression level of DNMT3B in clinical tumor tissues, we collected clinical tissue samples from 80 patients with a history of colorectal cancer surgery at Northern Jiangsu People’s Hospital. Tissue microarrays were constructed, and immunohistochemical staining was performed. DNMT3B was significantly overexpressed in tumor tissues compared to adjacent nontumor tissues ( Figure 1C). Additionally, fresh tissue samples were collected for protein extraction, and the western blot analysis results demonstrated high expression of DNMT3B in the tumor tissues ( Figure 1D,E).

To further investigate the role of DNMT3B in tumor progression, we examined the expression level of DNMT3B in common CRC cell lines. The results revealed that compared to that in the colon epithelial cell line NCM460, the mRNA expression of *DNMT3B* was elevated in the CRC cell lines SW620, HCT116, COLO205, and RKO ( Figure 1F). DNMT3B exhibited a similar trend at the protein level ( Figure 1G). Our follow-up data suggested that patients with high expression of DNMT3B exhibited a shorter survival duration than those with low expression of DNMT3B ( Figure 1H).

### DNMT3B is highly expressed in colorectal cancer

DNMT3B-positive staining is challenging to detect in normal tissues but readily observable in colorectal cancer tissues. Using ROC analysis, an IHC score of 5 served as the cut-off point to distinguish between high and low expression levels of DNMT3B. The rate of high DNMT3B expression in colorectal cancer tissue was 80%, which was significantly greater than that in normal tissue ( Table 1). We examined the correlation between DNMT3B expression and the clinicopathological characteristics of patients with CRC. High expression of DNMT3B significantly correlated with T stage, lymph node metastasis, tumor size, and distant metastasis but showed no significant associations with age, BMI, or gender ( Table 2).

**Table:** **Table 1** DNMT3B expression levels in colorectal cancer detected by IHC

Types	*n*	DNMT3B		*P*-value
Low expression	High expression
Tumor tissues	40	8 (20.0%)	32 (80.0%)		0.001
Normal tissues	40	22 (55.0%)	18 (45.0%)

**Table:** **Table 2** The correlation between DNMT3B expression and clinicopathological characters in colorectal cancer

Types	Low expression ( *n*=30)	High expression( *n*=50)	*P*-value
Gender Male [ *n* (%)] Female [ *n* (%)]	10 (12.5%) 20 (25.0%)	21 (26.3%) 29 (36.3%)	0.441
Age (SD)	59.97 (13.4)	58.92 (10.8)	0.702
BMI (kg/m ^2^) (SD)	23.9 (2.7)	24.5 (3.5)	0.448
T [ *n* (%)] 1 2 3 4	2 (2.5%) 15 (18.8%) 13 (16.3%) 0	4 (5.0%) 21 (26.3%) 13 (16.3%) 12 (15.0%)	0.026
N [ *n* (%)] 0 1 2	16 (20.0%) 11 (13.8%) 3 (3.8%)	10 (12.5%) 26 (32.5%) 14 (17.5%)	0.006
M [ *n* (%)] 0 1 TNM [ *n* (%)]	25 (31.3%) 5 (6.3%)	31 (38.8%) 19 (23.8%)	0.044 0.018
1	12(15.0%)	8 (10.0%)	
2	4 (5.0%)	2 (2.5%)	
3	9 (11.3%)	21 (26.3%)	
4	5 (6.3%)	19 (23.8%)	
Tumor size (cm) (SD)	3.2 (1.0)	4.6 (1.4)	0.000

### PLCG2 is identified as a key downstream regulatory protein of DNMT3B in CRC

**Figure 2:**
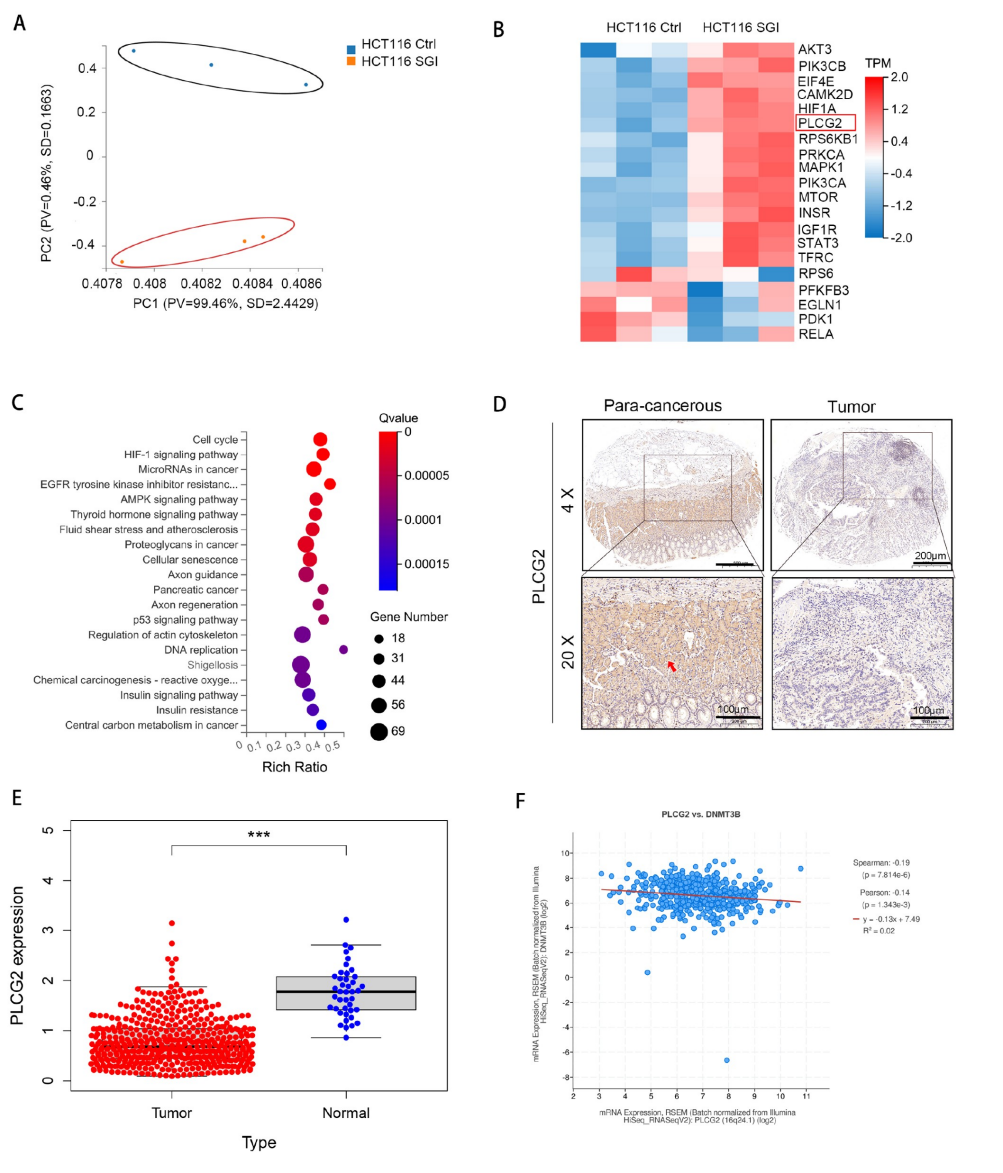
Figure 2 PLCG2 is identified as a key downstream regulatory protein of DNMT3B in CRC (A) Principal component analysis of the DEGs after SGI treatment for 24 h. (B) Heatmap analysis of HCT116 cells after SGI treatment for 24 h. (C) KEGG pathway enrichment analysis of the differentially expressed genes. (D) Immunohistochemical analysis of PLCG2 in paired noncancerous tissues and CRC tissues from patients. (E) The expression of PLCG2 in CRC was analyzed in the TCGA database. (F) The correlation between DNMT3B and PLCG2 expression in the TCGA database was analyzed. ***P<0.001.

To better delineate the downstream regulatory proteins of DNMT3B in CRC, we treated HCT1116 colon cancer cells with the DNMT3B inhibitor SGI-1027 (SGI) for 24 h, collected the cells, and performed transcriptomic sequencing. Principal component analysis (PCA) was conducted on the differentially expressed genes (DEGs) after SGI treatment. The results revealed distinct clustering of the DEGs in the SGI-treated group ( Figure 2A). Heatmap analysis of the differentially expressed genes revealed significant upregulation of the PLCG2 protein and notable differences in the PI3K-AKT signaling pathway ( Figure 2B). Subsequently, we performed KEGG pathway enrichment analysis on the differentially expressed genes, which indicated a significant impact on the tumor cell cycle ( Figure 2C). To investigate the clinical relevance of PLCG2, we conducted immunohistochemical staining of PLCG2 using tissue microarrays. The results showed stable expression of PLCG2 in normal tissues, whereas PLCG2 protein expression was decreased in colorectal cancer tissues ( Figure 2D). Similarly, we validated the expression of PLCG2 in the TCGA cohort and revealed a significant downregulation of PLCG2 expression in colorectal cancer tumor tissues ( Figure 2E).

Furthermore, we analyzed the correlation between DNMT3B and PLCG2 expressions in the TCGA database and revealed a significant negative correlation between DNMT3B and PLCG2 ( Figure 2F). These results suggested that DNMT3B may exert oncogenic effects by downregulating PLCG2.

### PLCG2 overexpression inhibits colorectal cell proliferation

**Figure 3:**
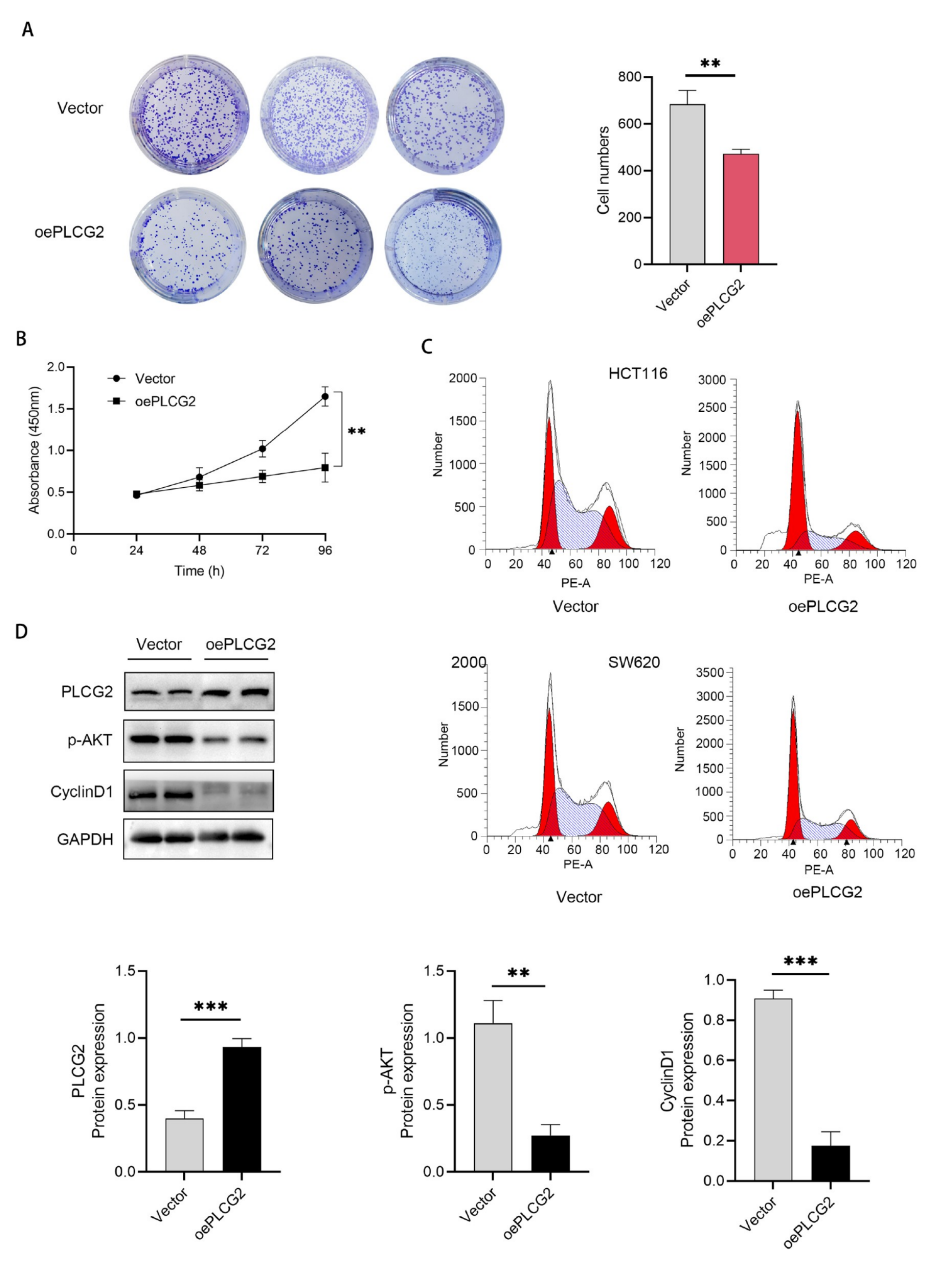
Figure 3 PLCG2 overexpression inhibits colorectal cell proliferation (A) Colony formation assay showing the proliferation of HCT116 cells after PLCG2 overexpression. (B) The proliferation of vector-transformed HCT116 cells and PLCG2-overexpressing cells was determined by CCK-8 assay. (C) The cell cycle distribution of vector-transformed HCT116 and PLCG2-overexpressing cells was detected by flow cytometry. (D) The protein level was detected by western blot analysis in HCT116 cells. **P<0.01, ***P<0.001.

To determine the role of PLCG2 in CRC, we constructed a lentiviral vector overexpressing PLCG2 and generated stable PLCG2-overexpressing cell lines. Plate colony formation assays were conducted on cells overexpressing PLCG2, revealing a reduction in tumor cell proliferation upon overexpression of the PLCG2 protein ( Figure 3A). CCK8 assays were performed on cells overexpressing PLCG2, which showed decreased proliferation at 48, 72, and 96 h compared to that of the control group ( Figure 3B).

Subsequently, flow cytometry analysis of the cell cycle distribution of SW620 and HCT116 cells overexpressing PLCG2 revealed a significant increase in the proportion of cells in the G1 phase after overexpression of PLCG2 ( Figure 3C). Protein was extracted from HCT116 cells overexpressing PLCG2 and subjected to western blot analysis, which revealed a significant decrease in the expression levels of the downstream proteins p-AKT and Cyclin D1 after overexpression of PLCG2 ( Figure 3D).

### DNMT3B inhibits PLCG2 transcription through methylation of the *PLCG2* promoter region

**Figure 4:**
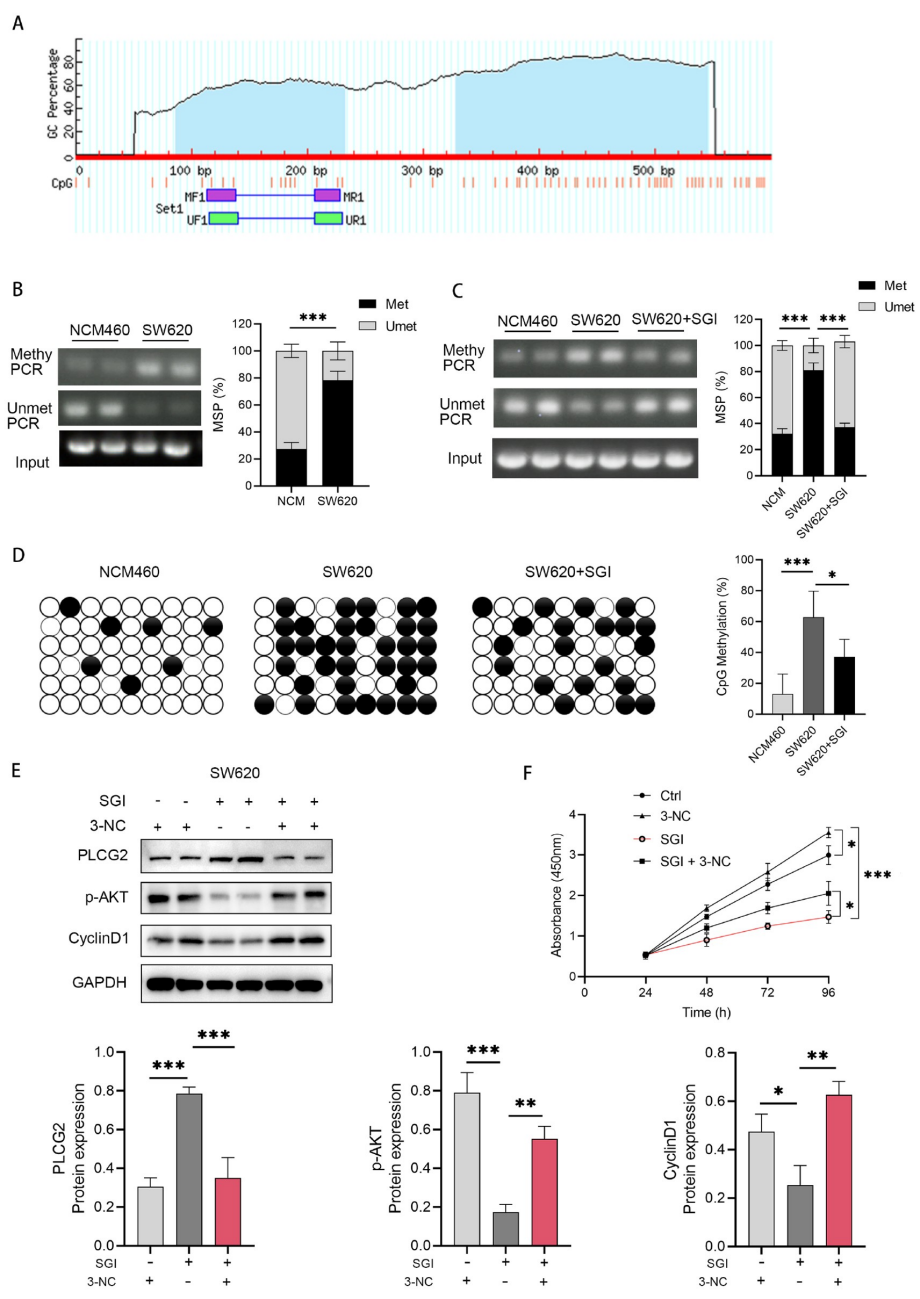
Figure 4 DNMT3B inhibits PLCG2 transcription through methylation of the *PLCG2* promoter region (A) The methylation sites in the PLCG2 promoter region were analyzed using the Methyprimor database. (B) Methylation-specific PCR amplification was conducted using methy and unmet primers designed for the PLCG2 promoter region after bisulfite modification. (C) MSP analysis of SW620 cells treated with SGI for 24 h. (D) BSP analysis of SW620 cells treated with SGI for 24 h. (E) In rescue experiments, PLCG2 protein level was increased after SGI treatment, while treatment with 3-NC reduced PLCG2 activity, resulting in the restoration of downstream p-AKT and CyclinD1 expressions. (F) The same methods were used for the CCK-8 experiments, in which cell viability was significantly decreased after 24 h of SGI treatment. Moreover, the proliferation capacity of cells treated with both SGI and 3-NC was greater than that of cells treated with SGI alone. *P<0.05, **P<0.01, ***P<0.001.

To elucidate the molecular mechanism by which DNMT3B regulates PLCG2, we analyzed the methylation sites in the *PLCG2* promoter region using the Methyprimor database. Unexpectedly, a significant CpG island was found 1000 bp upstream of the *PLCG2* transcription start site ( Figure 4A), indicating that DNMT3B may suppress PLCG2 expression through DNA methylation. Methylation-specific PCR amplification was conducted using methy and unmet primers designed for the *PLCG2* promoter region after bisulfite modification. Compared to normal colonic epithelial cells, colorectal cancer cell lines exhibited elevated levels of *PLCG2* methylation and decreased levels of non-methylation ( Figure 4B). To investigate the role of DNMT3B in methylation, cells were treated with the inhibitor SGI for 24 h, genomic DNA was extracted, and MSP experiments were performed. The results showed decreased methylation levels in colorectal cancer cells treated with SGI ( Figure 4C). To further validate our initial findings, we conducted additional bisulfite-specific PCR (BSP) methylation analysis on the identical region, adhering to the gold standard in methylation assessment. This analysis revealed a marked decrease in CpG methylation levels within colorectal cancer cells treated with SGI ( Figure 4D). Similarly, cell proteins were extracted using the same treatment method, and western blot analysis revealed that PLCG2 protein level increased after treatment with the DNMT3B inhibitor SGI ( Figure 4E).

PLCG2 is a substrate of Bruton’s tyrosine kinase (BTK), and decreased BTK level results in AKT phosphorylation. Elevated PLCG2 level leads to decreased p-AKT level and reduced CyclinD1 level, causing cell cycle arrest at the G1 phase ( Figure 4E). 3-NC is an inhibitor of PLCG2. In rescue experiments, PLCG2 protein level was increased after SGI treatment, while the 3-NC reduced PLCG2 activity, resulting in the restoration of downstream p-AKT and CyclinD1 expressions ( Figure 4E). The same methods were used for the CCK-8 experiments, in which cell viability was found to be significantly decreased after 24 h of SGI treatment. Moreover, the proliferation capacity of cells was greater in the group treated with both SGI and 3-NC than in the group treated with SGI alone ( Figure 4F).

### Overexpression of PLCG2 inhibits the formation of colorectal tumors in a xenograft model

**Figure 5:**
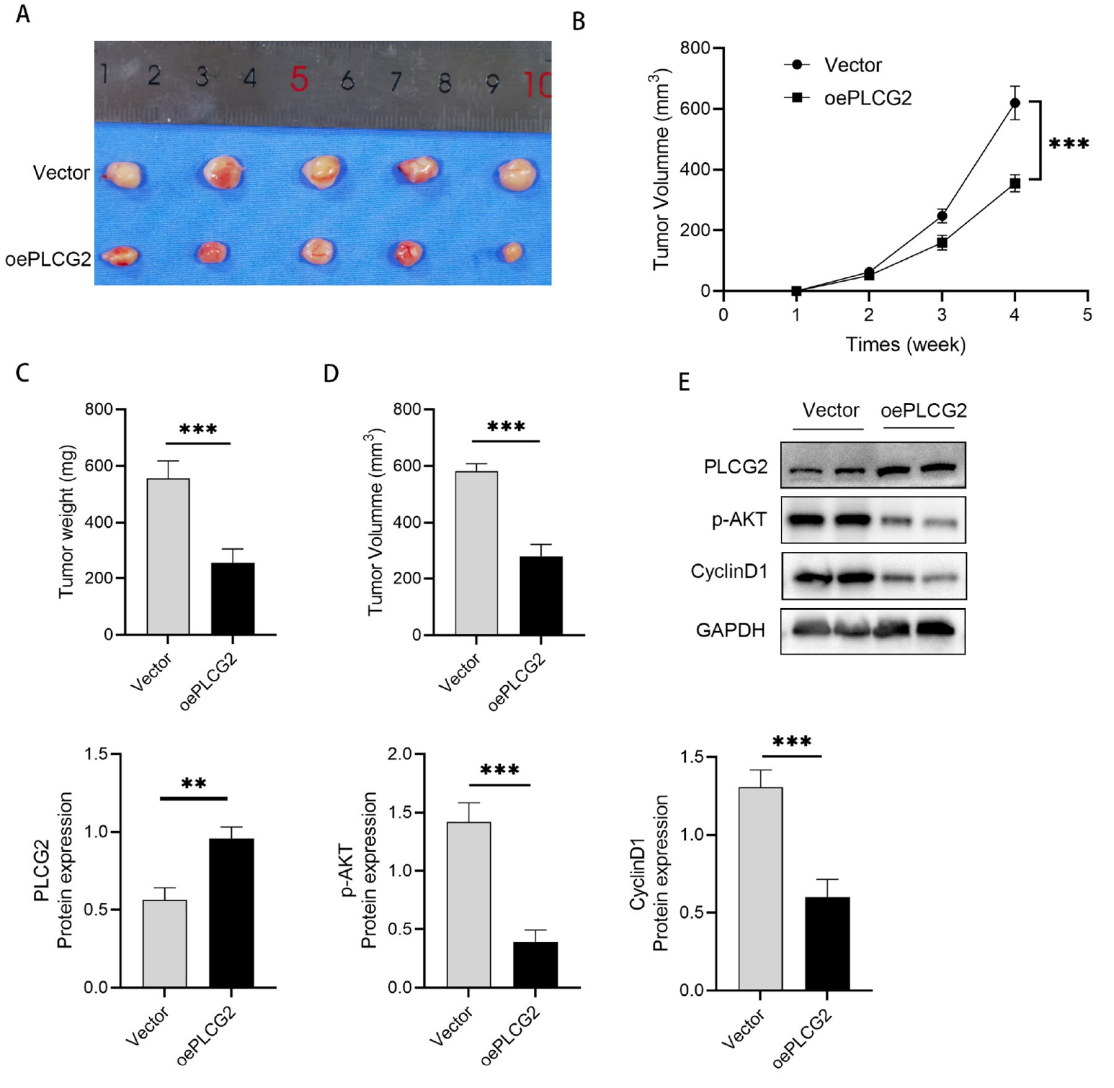
Figure 5 Effects of PLCG2 overexpression on tumorigenesis in nude mice *in vivo* (A) Xenograft models in nude mice were generated using HCT116 cells transfected with vector (n=5) or oePLCG2 (n=5). (B) Subcutaneous tumor growth curve in nude mice. (C) Measurement of the tumor weight of HCT116 cells transfected with the PLCG2 lentiviral OE vector after euthanasia. (D) Detection of the tumor volume of HCT116 cells transfected with the PLCG2 lentiviral OE vector after euthanasia. (E) The protein expressions in mouse tumor tissue were measured by western blot analysis. **P<0.01, ***P <0.001.

Immunodeficient BALB/c mice harboring HCT116 cells stably transfected with either the vector or oePLCG2 lentivirus were used to investigate the role of PLCG2 in colorectal cancer carcinogenesis *in vivo*. This was done to further validate whether PLCG2 functions in *in vivo* models. Four-week-old nude mice were subcutaneously injected with HCT116 vector cells (vector group) or PLCG2-overexpressing HCT116 cells (oePLCG2 group). A palpable mass emerged at the injection site one week postinjection. After 4 weeks, the tumors were harvested and examined ( Figure 5A). The largest and smallest diameters of the masses were measured weekly. As expected, beginning in the third week, compared with control treatment, PLCG2 overexpression significantly inhibited the growth of HCT116 tumors in mice ( Figure 5B). After the fourth week, the nude mice were euthanized, and tumor weights and volumes were assessed. The average weight of the oePLCG2 group (255.80±21.49 mg) was significantly lower than that of the vector group (555.80±27.70 mg) ( *P* <0.001; Figure 5C). Moreover, the average volume of the oePLCG2 group was markedly smaller than that of the vector group (279.20±18.59 vs 580.80±12.25 mm ^3^) ( *P* <0.001; Figure 5D). The protein expression in mouse tumor tissue was analyzed by western blot analysis. The results showed that the level of p-AKT CyclinD1 protein was significantly reduced after PLCG2 overexpression in the subcutaneous tumor tissues of the nude mice ( Figure 5E). These findings demonstrated that overexpression of PLCG2 effectively prevented the growth of colorectal cancer xenograft tumors *in vivo*.

## Discussion

CRC poses a significant threat characterized by a grim prognosis, delayed diagnosis, and insufficient treatment [1]. Early detection of CRC is infrequent due to vague symptoms that often manifest later in clinical progression. The pathogenesis of colorectal cancer is complex. For instance, aberrant regulation of transcription factors in colorectal cancer may result in abnormal gene expression, impacting processes such as cell proliferation, differentiation, and apoptosis [20]. Additionally, mitochondrial dysfunction and alterations in cellular energy metabolism may play a role in the onset of CRC [21]. Ubiquitination is a mechanism associated with protein degradation, and excessive ubiquitination can lead to abnormal degradation of crucial regulatory proteins, thereby influencing cellular functions [22]. The specific pathogenic mechanism of CRC remains incompletely elucidated. The mechanism involves the interplay of multiple factors, and ongoing research is continually advancing to provide a more comprehensive understanding of the disease formation process. Consequently, the identification of novel molecular pathways and potential therapeutic targets is imperative for mitigating the severity of CRC.

In this study, we revealed several significant discoveries that contribute to a deeper comprehension of the epigenetic mechanisms involved in CRC. The investigation of the relationship between DNMT3B and PLCG2 represents a crucial exploration in the field of molecular biology. DNMT3B, a vital member of the DNA methyltransferase family, is responsible for adding methyl groups to DNA molecules, influencing gene expression, and maintaining genomic stability [23]. On the other hand, PLCG2 is a key protein involved in cellular signaling pathways [ 24– 26]. Recent research has sparked an in-depth examination of the potential interactions and cross-pathways between DNMT3B and PLCG2, aiming to elucidate their mutual impact on cellular processes and functions. Understanding the relationship between these two molecules is important because it may clarify the intricate mechanisms governing gene regulation, epigenetic modifications, and signal transduction pathways. This study aimed to provide a comprehensive overview of the current research status regarding the relationship between DNMT3B and PLCG2. The exploration encompasses a detailed scrutiny of their individual functionalities, the molecular mechanisms underlying their interactions, and the biological consequences of their mutual influence.

The clinical relevance of DNMT3B in colorectal cancer has been a focus of research, with efforts directed towards assessing its potential as a diagnostic biomarker or therapeutic target [ 27– 29]. The development of antitumor agents has consistently been a central theme in clinical research [30]. In this domain, the focus of these endeavors is directed towards promoting the development of innovative drugs, with the aim of providing more effective and targeted therapeutic options for cancer patients. The exploration of DNMT3B inhibitors and their efficacy in mitigating colorectal cancer progression further signifies the translational implications of this research.

DNA methylation in the promoter region of *PLCG2* is known to influence its expression level. Aberrant methylation patterns of this gene have been associated with dysregulated signaling pathways and cellular functions in various cancers, including colorectal cancer. Alterations in DNA methylation within the regulatory regions of *PLCG2* may lead to changes in its expression level. This, in turn, can affect downstream signaling cascades and contribute to the progression of colorectal cancer. Association with Tumor Suppression: Studies suggest that DNA methylation-mediated silencing of *PLCG2* may interfere with its tumor-suppressive functions. Understanding the specific CpG sites involved and the extent of methylation alterations is crucial for deciphering their role in colorectal carcinogenesis [ 31– 33].

DNA methylation patterns in *PLCG2* may serve as potential diagnostic biomarkers for colorectal cancer. Analyzing these epigenetic modifications could aid in early detection and risk stratification. The correlation between the DNA methylation status of *PLCG2* and clinical outcomes could establish its utility as a prognostic indicator. Stratifying patients based on these epigenetic profiles may guide personalized treatment approaches.

As research on DNA methylation in *PLCG2* has advanced, further investigations are warranted to unravel the complexities of its epigenetic regulation, interactions with other signaling pathways, and functional consequences in colorectal cancer. The integration of these findings into clinical practice holds the potential to enhance diagnostic accuracy, prognostic precision, and the development of targeted epigenetic therapies for colorectal cancer patients.

In summary, PLCG2 has been identified as a key downstream regulatory protein of DNMT3B in CRC. DNMT3B inhibits *PLCG2* transcription through methylation of the *PLCG2* promoter region. DNMT3B controls CRC cell proliferation through PLCG2, which is useful for developing therapeutic approaches that target PLCG2 expression for the treatment of CRC.

## References

[1] Siegel RL, Miller KD, Jemal A (2019). Cancer statistics, 2019. CA Cancer J Clin.

[2] Lin W, Li C, Clement EA, Brown CJ, Raval MJ, Karimuddin AA, Ghuman A (2024). Surgical outcomes in total neoadjuvant therapy for rectal cancer versus standard long-course chemoradiation. Ann Surg.

[3] Reif de Paula T, Keller DS (2023). A national evaluation of adjuvant chemotherapy in pT4N0M0 colon cancer from the national cancer database. J Natl Cancer Inst.

[4] Yates J, Schaufelberger H, Steinacher R, Schär P, Truninger K, Boeva V (2024). DNA-methylation variability in normal mucosa: a field cancerization marker in patients with adenomatous polyps. J Natl Cancer Inst.

[5] Lykoskoufis NMR, Planet E, Ongen H, Trono D, Dermitzakis ET (2024). Transposable elements mediate genetic effects altering the expression of nearby genes in colorectal cancer. Nat Commun.

[6] Li Y, Xu J, Chen C, Lu Z, Wan D, Li D, Li JS (2024). Multimodal epigenetic sequencing analysis (MESA) of cell-free DNA for non-invasive colorectal cancer detection. Genome Med.

[7] AL-Jumaili MMO (2024). Transcription silencing and CpGs hypermethylation as therapeutic gene editing in clinical colorectal adenocarcinoma repression. Korean J Gastroenterol.

[8] Nagasaka T, Sasamoto H, Notohara K, Cullings HM, Takeda M, Kimura K, Kambara T (2004). Colorectal Cancer With Mutation in
*BRAF*,
*KRAS*, and wild-type with respect to both oncogenes showing different patterns of DNA methylation. J Clin Oncol.

[9] Grady WM, Yu M, Markowitz SD (2021). Epigenetic alterations in the gastrointestinal Tract: current and emerging Use for biomarkers of cancer. Gastroenterology.

[10] Dickinson BT, Kisiel J, Ahlquist DA, Grady WM (2015). Molecular markers for colorectal cancer screening. Gut.

[11] Yu H, Wang X, Bai L, Tang G, Carter KT, Cui J, Huang P (2023). DNA methylation profile in CpG-depleted regions uncovers a high-risk subtype of early-stage colorectal cancer. J Natl Cancer Inst.

[12] Li H, Yuan Y, Dong H, Wang T, Zhang D, Zhou L, Chen L (2023). Foxo3a-mediated DNMT3B impedes cervical cancer cell proliferation and migration capacities through suppressing PTEN promoter methylation. J Invest Surg.

[13] So JY, Yang HH, Park WY, Skrypek N, Ishii H, Chen JM, Lee MP (2022). DNA methyltransferase 3B-mediated intratumoral heterogeneity and therapeutic targeting in breast cancer recurrence and metastasis. Mol Cancer Res.

[14] Gui T, Liu M, Yao B, Jiang H, Yang D, Li Q, Zeng X (2021). TCF3 is epigenetically silenced by EZH2 and DNMT3B and functions as a tumor suppressor in endometrial cancer. Cell Death Differ.

[15] Wong CC, Kang W, Xu J, Qian Y, Luk STY, Chen H, Li W (2019). Prostaglandin E(2) induces DNA hypermethylation in gastric cancer
*in vitro* and
*in vivo*. Theranostics.

[16] Hu N, Wang F, Sun T, Xu Z, Zhang J, Bernard D, Xu S (2021). Follicular Lymphoma-associated BTK mutations are inactivating resulting in augmented AKT activation. Clin Cancer Res.

[17] Mandal S, Bandyopadhyay S, Tyagi K, Roy A (2021). Recent advances in understanding the molecular role of phosphoinositide-specific phospholipase C gamma 1 as an emerging onco-driver and novel therapeutic target in human carcinogenesis. Biochim Biophys Acta (BBA)-Rev Cancer.

[18] Li H, Brewer G, Ongo G, Normandeau F, Omeroglu A, Juncker D (2017). Immunohistochemistry microarrays. Anal Chem.

[19] Love MI, Huber W, Anders S (2014). Moderated estimation of fold change and dispersion for RNA-seq data with DESeq2. Genome Biol.

[20] Du L, Liu N, Jin J, Cao M, Sun Y, Gao X, Ruan B (2022). ZNF3 regulates proliferation, migration and invasion through MMP1 and TWIST in colorectal cancer. Acta Biochim Biophys Sin.

[21] Li J, Zheng W, Wu J, Zhang J, Lv B, Li W, Liu J (2023). CPT1C-mediated fatty acid oxidation facilitates colorectal cancer cell proliferation and metastasis. Acta Biochim Biophys Sin.

[22] Wang T, Jin C, Yang P, Chen Z, Ji J, Sun Q, Yang S (2023). UBE2J1 inhibits colorectal cancer progression by promoting ubiquitination and degradation of RPS3. Oncogene.

[23] Reik W, Dean W, Walter J (2001). Epigenetic reprogramming in mammalian development. Science.

[24] Mao D, Epple H, Uthgenannt B, Novack DV, Faccio R (2006). PLCgamma2 regulates osteoclastogenesis via its interaction with ITAM proteins and GAB2. J Clin Invest.

[25] Mueller H, Stadtmann A, Van Aken H, Hirsch E, Wang D, Ley K, Zarbock A (2010). Tyrosine kinase Btk regulates E-selectin–mediated integrin activation and neutrophil recruitment by controlling phospholipase C (PLC) gamma2 and PI3Kgamma pathways. Blood.

[26] Krepischi ACV, Maschietto M, Ferreira EN, Silva AG, Costa SS, da Cunha IW, Barros BDF (2016). Genomic imbalances pinpoint potential oncogenes and tumor suppressors in wilms tumors. Mol Cytogenet.

[27] Rhee I, Bachman KE, Park BH, Jair KW, Yen RWC, Schuebel KE, Cui H (2002). DNMT1 and DNMT3b cooperate to silence genes in human cancer cells. Nature.

[28] Karpf AR, Matsui S (2005). Genetic disruption of cytosine DNA methyltransferase enzymes induces chromosomal instability in human cancer cells. Cancer Res.

[29] Wendt MK, Johanesen PA, Kang-Decker N, Binion DG, Shah V, Dwinell MB (2006). Silencing of epithelial CXCL12 expression by DNA hypermethylation promotes colonic carcinoma metastasis. Oncogene.

[30] Wu Y, Pi D, Zhou S, Yi Z, Dong Y, Wang W, Ye H (2023). Ginsenoside Rh3 induces pyroptosis and ferroptosis through the Stat3/p53/NRF2 axis in colorectal cancer cells. Acta Biochim Biophys Sin.

[31] Issa JPJ, Vertino PM, Wu J, Sazawal S, Celano P, Nelkin BD, Hamilton SR (1993). Increased cytosine DNA-methyltransferase activity during colon cancer progression. JNCI J Natl Cancer Inst.

[32] Schmutte C, Yang AS, Beart RW, Jones PA (1995). Base excision repair of U:G mismatches at a mutational hotspot in the p53 gene is more efficient than base excision repair of T:G mismatches in extracts of human colon tumors. Cancer Res.

[33] Bhandari YR, Krishna V, Powers R, Parmar S, Thursby SJ, Gupta E, Kulak O (2023). Transcription factor expression repertoire basis for epigenetic and transcriptional subtypes of colorectal cancers. Proc Natl Acad Sci USA.

